# Mechanism of antibacterial action of the alcoholic extracts of *Hemidesmus indicus* (L.) R. Br. ex Schult, *Leucas aspera* (Wild.), *Plumbago zeylanica* L., and *Tridax procumbens* (L.) R. Br. ex Schult

**DOI:** 10.3389/fmicb.2015.00577

**Published:** 2015-06-09

**Authors:** Kongari Saritha, Angireddy Rajesh, Khanapur Manjulatha, Oruganti H. Setty, Suresh Yenugu

**Affiliations:** ^1^Department of Biochemistry, University of Hyderabad, Hyderabad, India; ^2^Department of Animal Biology, University of Hyderabad, Hyderabad, India

**Keywords:** herbal products, antibacterial activity, membrane permeabilization, flow cytometry

## Abstract

Herbal products derived from *Hemidesmus indicus* (L.) R. Br. ex Schult, *Leucas aspera* (Wild.), *Plumbago zeylanica* L., and *Tridax procumbens* (L.) R. Br. ex Schult. are widely used in traditional medicine. Though the extracts of these plants were found to be antimicrobial in nature and have the potential to be used in clinics, the mechanism of action of is not reported. The ethanolic extracts of *Hemidesmus indicus* (L.) R. Br. ex Schult, *Hemidesmus indicus* ethanolic extract (HIEE), *Leucas aspera* (Wild.), *Leucas aspera* ethanolic extract (LAEE), *Plumbago zeylanica* L., *Plumbago zeylanica* ethanolic extract (PZEE), and *Tridax procumbens* (L.) R. Br. ex Schult, *Tridax procumbens* ethanolic extract (TPEE) were tested for their antibacterial activity against *E. coli*. Antibacterial activity was analyzed by CFU assay and the effect on the bacterial membrane by fluorescence activated cell sorting and scanning electron microscopy. LAEE, PZEE, and HIEE displayed potent bacterial killing activity in a time and concentration dependent manner. TPEE did not display appreciable antibacterial activity. The antibacterial action involved disruption of membrane potential, inner membrane permeabilization, blebbing and leakage of cellular contents. Our results contribute to the understanding of the antibacterial mechanism of alcoholic extracts of the medicinal plants used in this study.

## Introduction

Complementary and alternative forms of medicine (CAM) have been practiced through out the world for centuries. Majority of these practices involve the use of crude plant extracts or purified products from different parts of plants and is dependent on the biodiversity available within the regions, thereby forming a local heritage (Purohit and Vyas, 2004a). In India, CAM is practiced for more than 5,000 years in different forms such as Ayurveda, Sidda, Homeopathy, and Unani and these are licensed by the Government. In the recent years, treatment strategies that involve use of natural products derived from plants has gained importance and this is primarily to overcome the side effects of allopathic forms of medicine. Besides this, multidrug resistance by pathogens to currently used antibiotics triggered the search for identifying natural antimicrobial products that are effective in combating infections (Cowan, 1999). The World health organization (WHO) estimates that the traditional systems of medicines are accepted by almost 80% of the population throughout the world for their primary health needs (Purohit and Vyas, 2004b). Further, the National Health Interview Survey (NHIS) conducted by the Centers for Disease Control and Prevention (CDC) in 2007 indicate that around 40% of adults in the United States used some form of complementary and alternative medicine, indicating that such medical practices are prevalent even in the developed nations (Barnes et al., 2008). Hence, CAM is accepted as one of the “mainstream” health care practices. Medicinal plants continue to play an important role in CAM treatment strategies since they produce a wide variety of natural compounds with high therapeutic value (Nostro et al., 2000). Examples include cocaine, codeine, digitoxin, galantamine, quinine, and morphine, which are widely used in many clinical conditions (Newman et al., 2003; Butler, 2004). Similarly, certain plant compounds such as arteether, nitisinone, and tiotropium are currently involved in late-phase clinical trials (Balunas and Kinghorn, 2005).

*Hemidesmus indicus* (L.) R. Br. ex Schult (HI; Family: Apocynaceae) is a medicinally important species and the extracts obtained from various parts of the plant were found to be diaphoretic, demulcent and antimicrobial (Jain and Basal, 2003). *Leucas aspera* (Wild.) (LA; Family: Lamiaceae) is an Indian herbal plant, whose extracts have been shown to have antinociceptive, antidiabetic, antimicrobial, antioxidant, and cytotoxic properties (Ali et al., 2001; Ravikumar et al., 2005; Bhagwat et al., 2008; Prajapati et al., 2010). *Plumbago zeylanica* L. (PZ; Family: Plumbaginaceae) is one of the most widely used medicinal plant because of its antimitotic, antibacterial, antinematicidal and anticancer properties (Pant et al., 2012). *Tridax procumbens* (L.) R. Br. ex Schult. (TP; Family: Asteraceae) is also a medicinally important plant used for the treatment of liver disorders, diarrhea wound healing, diabetes and infections Though the antimicrobial activity of the above mentioned plants and other medicinally important plants were reported (Cowan, 1999; Araujo et al., 2014; Choi et al., 2014; Marinas et al., 2014; Melo et al., 2014), a systematic study on the kinetics of bacterial killing and the mechanism of action have not yet been investigated. It is well known that the general mechanism of antimicrobial agents involves bacterial membrane permeabilization or disruption of membrane potential or inhibition of macromolecular synthesis (Zasloff, 2002). On the other hand, conventionally used antibiotics exert their action on microbes by influencing cell wall, protein and nucleic acid syntheses and metabolite activity. To overcome the use of increasing doses of antibiotics, treatment strategies that involve the use of additional antimicrobial agent(s) along with antibiotics has gained importance. It would be advantageous if the mechanism of action of the additional antimicrobial agent is different from that of the antibiotic used. Plant extracts or active principles of herbal products being non toxic to the mammalian cells would be the best choice to be combined with antibiotics. Hence, determining the mechanism of antimicrobial action of extracts obtained from medicinally important plants will help to design alternate or combinational treatment strategies to treat infections caused by antibiotic resistant bacteria. Hence, in this study, we determine the bacterial killing kinetics and the mechanism of action of the root extracts of HI and PZ and the whole plant extracts of LA and TP.

## Materials and Methods

### Plant Material

HI (roots), LA (whole plant), PZ (roots), and TP (whole plant) were collected during July—September, 2012 in and around University of Hyderabad campus (17.4600°N, 78.3350°E). A voucher specimen of HI (UoH/MDP/NA-00004), LA (UoH/MDP/NA-00002), PZ (UoH/MDP/NA-00003), and TP (UoH/MDP/NA-00001) has been preserved in our laboratory and in the herbarium for future reference. Precautions were taken to ensure the specimens are not damaged by insects. The different parts of plants were chosen as per their usage in the traditional system of medicine (THE AYURVEDIC PHARMACOPOEIA OF INDIA, Volumes 1–7 published by Government Of India Ministry Of Health And Family Welfare Department Of Ayush, New Delhi, India).

### Preparation of Extracts and Phytochemical Screening

The powdered plant material of HI (roots), LA (whole plant), PZ (roots), and TP (whole plant) were suspended in 95% ethanol, since it is a polar solvent. They were extracted using Soxhlet for 16–24 h or until the solvent was clear. Ethanol solvent used for extraction was recovered under reduced pressure (Buchii rotavapor). The residual extracts were weighed and reconstituted in phosphate buffered saline (PBS) at a concentration of 10 mg/ml. Required amount of this extract was added directly to the assays and the equivalent amount of PBS was added to the controls. The ethanolic extracts obtained from HI (roots), LA (whole plant), PZ (roots), and TP (whole plant) were labeled as HIEE, LAEE, PZEE, and TPEE.

Phytochemical screening was carried out using qualitative tests as described earlier (Sofowora, 1993; Harborne, 1998). All the extracts were diluted with distilled water in the ratio of 1:100 (w/v) except for the cardiac glycoside test. For the alkaloid detection, 5 ml of the extract was added to 2 ml of HCl. To this acidic medium, 1 ml of Dragendroff’s reagent was added. An orange or red precipitate produced immediately indicated the presence of alkaloids. To test the presence of flavonoids, to 1 ml of the extract, a few drops of dilute sodium hydroxide was added. The appearance of an intense yellow color that becomes colorless on addition of a few drops of dilute acid indicates the presence of flavonoids. Steroids were detected by adding 1 ml of the extract to 10 ml of chloroform and an equal volume of concentrated sulphuric acid. Presence of steroids is indicated by the appearance of red and green colors in the upper and sulphuric acids layers. For saponin detection, the extract was diluted with 20 ml of distilled water and was agitated in a graduated cylinder for 15 min. The formation of 1 cm layer of foam showed the presence of saponins. Phenols were detected by warming 2 ml of the extract to 45–50°C followed by addition of 2 ml of 3% FeCl_3_. Formation of green or blue color indicated the presence of phenols.

### Determination of Total Phenolic and Flavonoid Content

The amount of total soluble phenolic content in the plant extracts (HIEE, LAEE, PZEE, and TPEE) was determined as described earlier (Singleton et al., 1999; Yang et al., 2007). Briefly, an aliquot (10 μl) of each extract was mixed with 1 ml of dd H_2_O and 100 μl of Folin–Ciocalteu’s phenol reagent. 300 μl of 20% Na_2_CO_3_ solution was added to the mixture followed by incubating at room temperature in the dark for 2 h and 30 min. The absorbance against a blank was measured at 735 nm using UV–Vis spectrophotometer. Gallic acid was used to prepare a standard curve. The total phenolic content was measured as Gallic acid equivalents [mg GAE/gram of dry weight (DW)] and the values were presented as means of triplicate analysis.

Total flavonoid content of the extracts was estimated using standard protocols (Dewanto et al., 2002; Barreira et al., 2010). An aliquot (20 μl) of each extract was mixed with 500 μl of double distilled water and 30 μl of 5% NaNO_2_ solution. After proper mixing and 10 min incubation, 60 μl of 10% AlCl_3_ solution was added. Subsequently, 350 μl of 1 M NaOH and 40 μl of ddH_2_O were added to make the final volume to 1 ml. Samples were further incubated for 15 min at room temperature and the absorbance of the samples was measured at 510 nm against blank. Quercetin was used as standard reference. The total flavonoids were determined as Quercetin equivalents (mg QE)/g of DW and the values were expressed as means of triplicate analysis.

### Antibacterial Activity

The colony forming units (CFU) assay was employed to test the antibacterial activity as described by (Rajesh and Yenugu, 2012). *E. coli* XL-1 Blue obtained commercially (Stratagene, La Jolla, CA, USA) was used to determine the antibacterial activity of the plant extracts. The bacterial cells were cultured in Luria–Bertani medium at 37°C. Bacterial cells grown to mid-log phase (A600 = 0.4–0.5) were diluted with 10 mM sodium phosphate buffer (pH 7.4). Varying concentrations (10–500 μg/ml) of TPEE, LAEE, HIEE, and PZEE were added to approximately 2 × 10^6^ CFU/ml of bacteria and incubated at 37°C for 30–180 min. After incubation, the assay mixtures were diluted (1:100 and 1:1000) with 10 mM sodium phosphate buffer (pH 7.4) and 50 μl of each was spread on a Luria–Bertani agar plate and incubated at 37°C overnight to allow full colony development. The resulting colonies were hand counted and plotted as log CFU/ml. The antibacterial activity was considered to be potent only when there were more than two logs of reduction in bacterial count.

### Determination of Membrane Potential

Membrane potential across the bacterial membrane was measured as described previously (Novo et al., 2000; Yeh et al., 2012). The effect of plant extracts on the membrane potential of bacteria was measured by using two different dyes, namely, 3,3′-diethyloxacarbocyanine iodide, DiOC_2_(3) and TO-PRO-3. DiOC_2_(3) can emit green and red fluorescence. The green fluorescence intensity of this dye is largely dependent on the membrane potential, where as the red fluorescence is dependent on the cell size and membrane potential. The ratio of red/green fluorescence gives a measure of the altered membrane potential. In order to eliminate dead cells that may also accumulate DiOC_2_(3) due to altered membrane permeability, TO-PRO-3, a fluorescent dye that specifically stains dead bacterial cells was included in the assay. Cells that display fluorescence signal of TO-PRO-3 (far red>695 nm) were excluded from the assay so that the membrane potential is measured only in the live cells. *E. coli* were treated with 100 μg/ml of LAEE or HIEE or PZEE for 2 h followed by 30 μM DiOC_2_(3) and 100 nM TOPRO-3 for 30 min. Fluorescence intensity was measured using fluorescein isothiocyanate (FITC) and Texas Red channels for green and red fluorescence respectively, whereas TOPRO-3 fluorescence was measured with PerCP-Cy6 channel in fluorescence activated cell sorting (FACS) life science research (LSR) Fortessa flow cytometer (BD Biosciences). Carbonyl cyanide *m*-chlorophenylhydrazone (CCCP), a proton ionophore was used as a positive control. The ratio of red/green mean fluorescence intensity was calculated to determine the membrane potential.

### Membrane Permeabilization

To determine the effects of plant extracts on bacterial membrane integrity, FACS analyses was carried out as described earlier with slight modifications (Mangoni et al., 2004). Bacterial membranes under normal conditions are impermeable to FITC, a fluorescent dye. Membrane destabilization or permeabilization by antibacterial agents would allow the entry of FITC into the cells. Measuring the fluorescence intensity emitted by FITC present inside the bacterial cell gives a direct measure of the extent of membrane permeabilization caused by the test compound. Generally, the fluorescence intensity of FITC inside the bacterial cells is monitored by confocal microscopy and this is only a qualitative measure. To allow quantification, we slightly modified the protocol in which the fluorescence intensity in the bacterial cells was analyzed using a flow cytometer. Briefly, log-phase *E. coli* were incubated for 2 h with 100 μg/ml of LAEE or HIEE or PZEE in growth medium containing FITC. The cells were then washed thrice to remove the excess FITC present in the medium. Mean fluorescence intensity was measured in the cells using FITC channel in a LSR Fortessa flow cytometer (BD Biosciences). Tetracycline (5 μg/ml), a potent membrane permeabilizing agent was used as the positive control.

### Scanning Electron Microscopy

The ability of plant extracts to alter the membrane integrity of *E. coli* was visualized using scanning electron microscopy as described earlier (Yenugu et al., 2006). Approximately 10^8^ CFU/ml of *E. coli* resuspended in 10 mM sodium phosphate buffer (pH 7.4) and treated with 200 μg/ml of the plant extract and incubated for 30 min at 37°C. The bacterial cells were washed and resuspended in 10 mM sodium phosphate buffer (pH 7.4) and fixed with an equal volume of 4% glutaraldehyde. Fixed cells were vacuum filtered onto a 0.1 μm polycarbonate membrane filter and dehydrated through a graded series of ethanol (30, 50, 75, and 100%). The filters were then dried and mounted on aluminum specimen supports and coated with 15 nm thickness of gold-palladium metal (60:40 Alloys). Samples were examined on a Cambridge Stereoscan 200 scanning electron microscope using an accelerating voltage of 20 kV.

### Statistical Analysis

Statistical analyses were performed using one way ANOVA and Holm-Sidak test available in Sigma Plot software (SPSS Inc., Chicago, IL, USA). Values shown are mean ± SD.

## Results

### Phytochemical Screening

The phytochemical composition of the plant extracts used in this study (HIME, LAEE, PZEE, and TPEE) was evaluated to determine the variations in the composition of alkaloid, flavonoid, steroid, saponin, and phenolic content. Phytochemical tests revealed that alkaloids were not present in all the extracts analyzed (Table 1). However, the other class of compounds, i.e., steroids, phenolics, flavonoids, and saponins were detected. Further analyses were carried out to determine the flavonoid and phenolic content (Table 1). Flavonoid content (mg GAE/g) was found to be highest (845 ± 0.061) in PZEE followed by LAEE (610 ± 0.011), TPEE (340 ± 0.024), and HIME (75 ± 0.022). On the same lines, the total phenolic content (mg QE/g) was found to be highest in PZEE (552 ± 0.08) followed by LAEE (417 ± 0.09), TPEE (227 ± 0.12), and HIME (18.97 ± 0.16).

**TABLE 1:** **Phytochemical properties, flavonoid and phenolic composition of plant extracts used in this study**.

**Extract**	**Physical appearance **	**Phytochemical tests**					**Flavonoid Content (mg QE/g)**	**Total phenolic content (mg GAE/g)**
		**Alkaloid**	**Steroids**	**Phenolic**	**Flavonoid**	**Saponin**		
TPEE	Greenish sticky semisolid	–	+	+	+	+	340 ± 0.024	227 ± 0.12
LAEE	Greenish sticky semisolid	–	+	+	+	+	610 ± 0.011	417 ± 0.09
HIEE	Dark brownish sticky semisolid	–	+	+	+	+	75 ± 0.022	8.97 ± 0.16
PZEE	Reddish brown syrupy	–	+	+	+	+	845 ± 0.061	552 ± 0.08

### Antibacterial Activity

**FIGURE 1:**
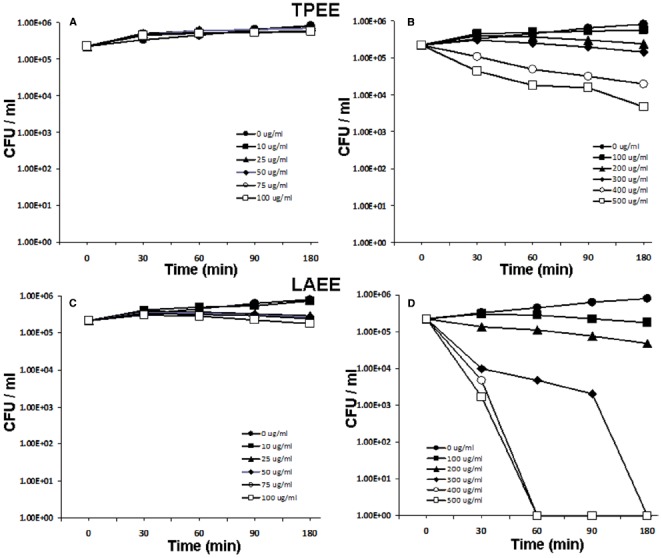
**Antibacterial activity of TPEE and LAEE.** *E. coli* were incubated with 0–100 μg/ml **(A)** or 100–500 μg/ml **(B)** TPEE or 0–100 μg/ml **(C)** or 100–500 μg/ml **(D)** LAEE for 0–180 mins. Bacterial count was analyzed by CFU assay. Values shown are mean ± SD.

**FIGURE 2:**
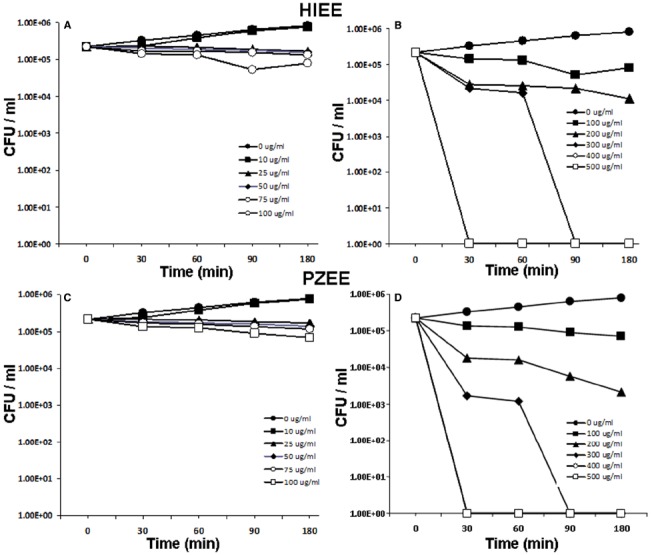
**Bacterial killing kinetics of HIEE and PZEE.** *E. coli* were incubated with 0–100 μg/ml **(A)** or 100–500 μg/ml **(B)** HIEE or 0–100 μg/ml **(C)** or 100–500 μg/ml **(D)** PZEE for 0–180 mins. Bacterial count was analyzed by CFU assay. Values shown are mean ± SD.

To assess the antibacterial property of TP, *E. coli* were treated with varying concentrations of with TPEE. At concentrations ranging from 10 to 100 μg/ml, bacterial killing was not evident (Figure 1A). When bacteria were incubated with higher concentrations ranging from 200 to 500 μg/ml, bacterial killing was observed with 400 and 500 μg/ml concentrations (Figure 1B). However, the decrease in bacterial count did not go beyond two log units, suggesting that TPEE may not possess appreciable antimicrobial activity. Antibacterial activity was not observed when *E. coli* were incubated with 10–100 μg/ml of LAEE (Figure 1C), but potent bacterial killing was evident at the higher concentrations in a dose and time dependent manner (Figure 1D). Similar bacterial killing kinetics was observed with HIEE (Figures 2A,B) and PZEE (Figures 2C,D). Complete bacterial killing was observed at the 300 μg/ml concentration for LAEE, HIME, and PZEE. However, the time taken to accomplish complete bacterial killing differed among the three alcoholic extracts, which was 180 min for LAEE and 90 min for both HIEE and PZEE. On the same lines, the time taken to accomplish complete bacterial killing by LAEE was 60 min at 400 and 500 μg/ml concentrations, whereas the same was 30 min for HIEE and PZEE at the same concentrations.

### Membrane Potential

**FIGURE 3:**
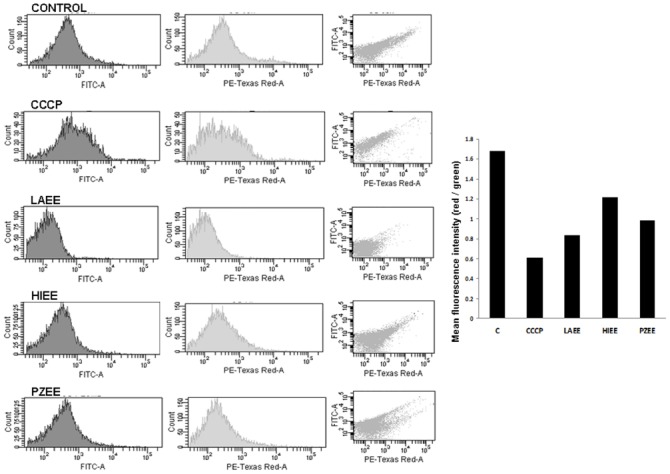
**Disruption of membrane potential by plant extracts.** *E. coli* treated with 100 μg/ml of LAEE or HIEE or PZEE or CCCP (15 μM) and incubated with 30 μM DiSO2(3). Red and green fluorescence intensity was measured using FITC and Texas red channels in a FACS fortessa flow cytometer. Intensity of red (left panel) and green fluorescence (middle panel) and cells exhibiting both the fluorescence intensities (left panel) are shown. Bar graph shows the membrane potential of *E. coli* without (control; C) or with CCCP or different plant extracts treatment.

In bacterial cells with intact cytoplasmic membrane, a difference of electric potential (membrane potential) exists across the membrane with negative charge in the interior and positive in the exterior. To determine whether the antibacterial action of LAEE or HIEE or PZEE involves disruption of bacterial membrane potential, the red and green fluorescence emitted by DiOC_2_(3) was measured. The ratio of red to green florescence gives and indication of the altered membrane potential. The red and green fluorescence exhibited by the bacterial cells is shown on the X and Y axis respectively (third panel of Figure 3). Treatment of *E. coli* with the ionophore CCCP resulted in decreased ratio of red to green fluorescence indicating a reduction in membrane potential (Figure 3). Treatment with LAEE or HIEE or PZEE also caused a decrease in the ratio of green to red fluorescence (Figure 3), suggesting that the mechanism of action involves disruption of membrane potential. Among the three ethanolic extracts tested, LAEE seems to be more potent followed by PZEE and HIEE.

### Membrane Permeabilization

**FIGURE 4:**
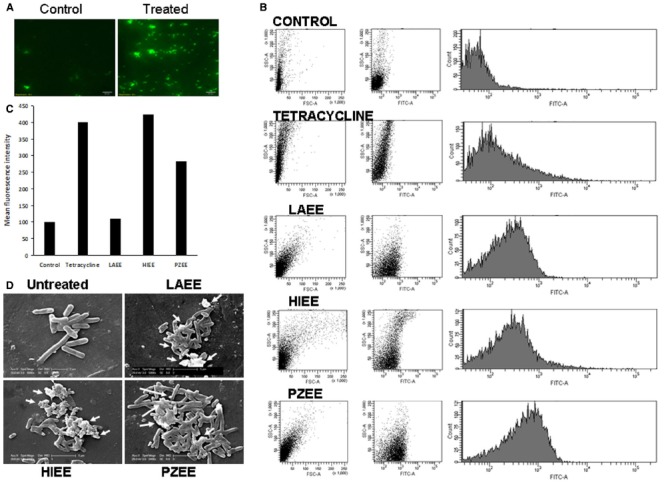
**Inner membrane permeabilization induced by plant extracts.** *E. coli* were incubated with 100 μg/ml of LAEE or HIEE or PZEE or tetracycline (5 μg/ml) for 2 h followed by incubation with 5 μg/ml of FITC. **(A)** Representative fluorescence microscopy images of *E. coli* showing influx of FITC with or without PZEE treatment. **(B)** FACS cytograms of control, LAEE or HIEE or PZEE treated *E. coli*. Left panel indicates forward and side scatter measurement; middle panel indicates population of FITC positive cells; left panel indicates fluorescence intensity range of FITC positive cells. **(C)** Mean fluorescence intensity in *E. coli* treated with different plant extracts. **(D)** Scanning electron microscopy. Bacterial cells treated with 200 μg/ml of LAEE or HIEE or PZEE for 2 h were visualized under scanning electron microscope. Arrows indicate the damage observed.

Antibacterial agents, in general, act on the membranes of microbes to cause destabilization and permeabilization. Bacterial cells in general are impermeable to FITC, a fluorescent dye; but upon membrane destabilization or permeabilization, FITC can enter the cells. Intense fluorescence was observed in bacterial cells treated with PZEE (Figure 4A), suggesting inner membrane permeabilization. The ability and the extent of plant extracts to permeabilize *E. coli* membrane was assessed by FACS analyses (Figure 4B). The first and second panels show the side scatter and FITC fluorescence exhibited by the bacterial cells respectively, whereas the third panel displays the mean fluorescence intensity of FITC. To validate the assay, tetracycline, a membrane permeabilizing agent was used as a positive control. The mean fluorescence intensity was increased in cells treated with tetracycline (Figure 4C). Treatment with HIEE or PZEE also caused an increase in the fluorescence intensity indicating the entry of FITC into the cells, thus membrane permeabilization (Figure 4C). Among the two, HIME seems to be more a potent membrane permeabilization agent than PZEE is comparable with that of tetracycline. However, LAEE treatment did not cause an increase in mean fluorescence intensity.

### Scanning Electron Microscopy

Disruption of membrane integrity, results in leakage of cellular contents and death. In order to determine whether the plant extracts used in this study exert their antibacterial action by destabilizing the membrane, scanning microscopy was performed. Untreated *E. coli* displayed smooth and even membrane morphology. *E. coli* treated with HIEE or LAEE or PZEE showed distorted membrane morphology, blebbing of membrane, aggregation and leakage of cellular contents (Figure 4D). These results demonstrate that the plant extracts act on the membrane potential and elicit bacterial killing effects.

## Discussion

Multi drug resistance by pathogens to conventionally used antibiotics led to the need for identifying novel anti infectives with mechanisms of actions that cannot be circumvented by the microbes. Proteins, peptides and many other small molecules derived from animal and plant sources were found to be antimicrobial. However, the mechanism of action of the plant products has received little attention. Hence, in this study, we attempted to understand the structural changes that occur in *E. coli* due to the antimicrobial action of ethanolic extracts of HI, LA, TP and PA. HIEE, LAEE, and PZEE exhibited potent antimicrobial activity. The antimicrobial activity of extracts prepared from different parts of HI, LA, PA, and TP is reported (Malathy and Sini, 2009; Naqash and Nazeer, 2011; Chew et al., 2012; Jindal and Kumar, 2012). Our results are in agreement with the previously reported studies. Though the antimicrobial, plasmodicidal, and antileishmanial activities of TP were reported (Martin-Quintal et al., 2010; Appiah-Opong et al., 2011; Naqash and Nazeer, 2011), we did not observe appreciable antibacterial activity with the alcoholic extract of TP. In our study, we used the extract of the whole plant, whereas in previous studies leaf extract or flavonoids obtained from this plant were studied (Martin-Quintal et al., 2010; Appiah-Opong et al., 2011; Naqash and Nazeer, 2011). It is possible that the minimal antibacterial activity of TPEE observed in our study could be due to use of whole plant extract, which may have lesser concentration of the active compounds when compared to extracts obtained from a single organ of the plant.

The crude and partially purified extracts of plants tend to contain a mixture of molecules that vary in chemical structure and composition, which in turn may influence the biological actions. The flavonoid and total phenolic content differed among the plant extracts used in this study; with the highest content observed in PZEE. However, a correlation between the flavonoid or phenolic content and the antibacterial activity. However, we observed that PZEE that has the highest content was the most potent among the four extracts used. The antibacterial activity observed could also be due to the phytochemicals other than flavonoids.

Evaluating the time course killing of bacterial cells by antimicrobials is essential to speculate whether the antimicrobial action of a compound is instantaneous (affecting the membrane integrity) or time dependent (affecting the cellular processes). In order to determine the mode of action of the alcoholic extracts used in this study, the kinetics of bacterial killing at different concentrations was evaluated. The time dependent killing of *E. coli* by alcoholic extracts of the plants suggests that the antimicrobial action observed could be due to the effect on a variety of physiological factors inside the cell. Further studies are required to test the ability of the plant extracts to affect the cellular process such as inhibition of macromolecular synthesis.

Antimicrobial agents disrupt bacterial membrane integrity thereby affecting the membrane potential. In this study, we observed disruption of membrane potential by LAEE, HIEE, and PZEE. Further, influx of FITC into *E. coli*, was evident, indicating that HIEE and PZEE have the ability to permeabilize inner membrane of *E. coli*. Such a mechanism of action was not reported till date using whole plant extracts or compounds isolated from different parts of the plant. It is very interesting to note that LAEE, which exhibited potent antibacterial activity, induced membrane potential disruption, but failed to induce inner membrane permeabilization. It is possible that the antibacterial action of LAEE could be restricted to membrane potential disruption, whereas the action of HIEE and PZEE could involve both membrane potential disruption and membrane permeabilization. The membrane potential disrupting ability of the extracts of medicinally important plants is reported. For example, the methanolic extract of *Gracilaria tenuistipitata* significantly decreased the mitochondrial membrane potential [measured by the fluorescence intensity emitted by DiOC_2_(3)] in MEGT-treated Ca9-22 cancer cells (Yeh et al., 2012). The extracts of *Annona muricata* had the ability to disrupt mitochondrial potential to induce apoptosis in cancer cells (Pieme et al., 2014). Further, electron microscopy revealed membrane blebbing and leakage of bacterial contents when bacterial cells were treated with HIEE or LAEE or PZEE. It is widely believed that the antimicrobial action of a variety of proteins and small molecules result in formation of pores in the bacterial membranes and cause leakage of cellular contents (Yenugu et al., 2006). The ability of HIEE or LAEE or PZEE to cause leakage of cellular contents suggests that they act to cause pores in the bacterial membranes. The membrane damaging action of these extracts could be due to the detergent properties of phenols and flavonoids. However, other mechanisms such as inhibition of macromolecular synthesis that may operate during bacterial killing by these plant extracts needs further investigation.

In conclusion, we report that the ethanolic extracts of HI, LP, and PZ are antibacterial and their action is time and concentration dependent. The mechanism of action of involves disrupting the bacterial membrane potential, permeabilization and leakage of cellular contents. The antibacterial potential of these plant extracts can be exploited to treat infections in place of conventional antibiotics.

### Conflict of Interest Statement

The authors declare that the research was conducted in the absence of any commercial or financial relationships that could be construed as a potential conflict of interest.

## Abbreviations

CAM: Complementary and alternative forms of medicine
CCCP: Carbonyl cyanide *m*-chlorophenylhydrazone
DiOC2(3): 3,3′-diethyloxacarbocyanine iodide
FACS: fluorescence activated cell sorting
FITC: Fluorescein isothiocyanate
HI: *Hemidesmus indicus*
HIEE: *Hemidesmus indicus* ethanolic extract
LA: *Leucas aspera*
LAEE: *Leucas aspera* ethanolic extract
PZ: *Plumbago zeylanica*
PZEE: *Plumbago zeylanica* ethanolic extract
TP: *Tridax procumbens*
TPEE: *Tridax procumbens* ethanolic extract.
